# Therapeutic Potential and Pharmaceutical Development of a Multitargeted Flavonoid Phloretin

**DOI:** 10.3390/nu14173638

**Published:** 2022-09-02

**Authors:** Kartik T. Nakhate, Hemant Badwaik, Rajesh Choudhary, Kalyani Sakure, Yogeeta O. Agrawal, Charu Sharma, Shreesh Ojha, Sameer N. Goyal

**Affiliations:** 1 Department of Pharmacology, Shri Vile Parle Kelavani Mandal’s Institute of Pharmacy, Dhule 424001, Maharashtra, India; 2 Department of Pharmaceutical Chemistry, Shri Shankaracharya Institute of Pharmaceutical Sciences and Research, Bhilai 490020, Chhattisgarh, India; 3 Department of Pharmacology, Shri Shankaracharya College of Pharmaceutical Sciences, Bhilai 490020, Chhattisgarh, India; 4 Department of Pharmaceutics, Rungta College of Pharmaceutical Sciences and Research, Bhilai 490024, Chhattisgarh, India; 5 Department of Pharmaceutics, Shri Vile Parle Kelavani Mandal’s Institute of Pharmacy, Dhule 424001, Maharashtra, India; 6 Department of Internal Medicine, College of Medicine and Health Sciences, United Arab Emirates University, Al Ain P.O. Box 15551, United Arab Emirates; 7 Department of Pharmacology and Therapeutics, College of Medicine and Health Sciences, United Arab Emirates University, Al Ain P.O. Box 15551, United Arab Emirates

**Keywords:** phloretin, therapeutic potential, physicochemical properties, pharmaceutical development, toxicity

## Abstract

Phloretin is a flavonoid of the dihydrogen chalcone class, present abundantly in apples and strawberries. The beneficial effects of phloretin are mainly associated with its potent antioxidant properties. Phloretin modulates several signaling pathways and molecular mechanisms to exhibit therapeutic benefits against various diseases including cancers, diabetes, liver injury, kidney injury, encephalomyelitis, ulcerative colitis, asthma, arthritis, and cognitive impairment. It ameliorates the complications associated with diabetes such as cardiomyopathy, hypertension, depression, memory impairment, delayed wound healing, and peripheral neuropathy. It is effective against various microbial infections including *Salmonella typhimurium*, *Listeria monocytogenes*, *Mycobacterium tuberculosis*, *Escherichia coli*, *Candida albicans* and methicillin-resistant *Staphylococcus aureus*. Considering the therapeutic benefits, it generated interest for the pharmaceutical development. However, poor oral bioavailability is the major drawback. Therefore, efforts have been undertaken to enhance its bioavailability by modifying physicochemical properties and molecular structure, and developing nanoformulations. In the present review, we discussed the pharmacological actions, underlying mechanisms and molecular targets of phloretin. Moreover, the review provides insights into physicochemical and pharmacokinetic characteristics, and approaches to promote the pharmaceutical development of phloretin for its therapeutic applications in the future. Although convincing experimental data are reported, human studies are not available. In order to ascertain its safety, further preclinical studies are needed to encourage its pharmaceutical and clinical development.

## 1. Introduction

Flavonoids are biologically active molecules widely present in foodstuffs of herbal origin. Several health benefits are associated with the regular intake of flavonoids—in particular, decreased risk of various diseases [1]. Therefore, interest has been growing in dietary flavonoids, primarily in fruit- and vegetable-rich diets. Phloretin is a dihydrogen chalcone flavonoid naturally present in ample amounts in apples and strawberries [2]. This compound has been demonstrated to target multiple biochemical, cellular and molecular mediators that modulate intracellular signaling, which eventually results in multiple health and therapeutic benefits. In the field of cancer research, several investigators have extensively studied the therapeutic potential of phloretin [3,4,5,6,7,8,9,10]. In addition to this, phloretin has many pharmacological activities, such as antidiabetic [11,12,13,14,15,16], cardioprotective [14,17,18,19,20], hepatoprotective [21,22,23], anti-inflammatory [2,24,25,26,27,28], antioxidant [29,30,31,32,33,34,35], immunosuppressant [36], and antimicrobial [37,38,39,40,41,42]. Although of natural origin, the intraperitoneal and oral administration of phloretin at 2.4 mmol/kg dose produced 100% and 64% mortality, respectively, in mice with acetaminophen-induced hepatotoxicity. However, the lower doses were not found lethal [43]. Therefore, besides potential therapeutic activities, additional investigations are vital to explore the adverse effects of phloretin. It may be noted that phloretin (aglycone) is present with its glycone phlorizin, also called phloridzin (phloretin 2′-O-glucose), in its biological sources [11]. However, after oral administration, the lactase hydrolase enzyme metabolized phlorizin to phloretin and glucose in the epithelial brush border membrane of the small intestine. Thus, the biological activities of phlorizin might be a result of the activities of aglycone phloretin [44,45]. Although phlorizin exerts several biological effects like those of phloretin, its chronic oral administration in rats with type 2 diabetes caused a marked reduction in muscle mass/strength, and substantial osteoporotic changes [44].

Despite its reported benefits, the major issue with orally administered phloretin is its poor bioavailability (8.67%) [46]. The low water solubility of phloretin results in poor absorption and bioavailability [47,48]. Moreover, after absorption, phloretin was found to be rapidly eliminated from the body [49]. Therefore, because of its better bioavailability and lower toxicity, phlorizin—a phloretin glycoside—is frequently utilized as a substitute for phloretin in numerous commercial products [50]. The poor solubility of phloretin limits its usage in traditional drug delivery systems. To overcome these challenges, nanotechnology has been explored as one of the potential techniques that can significantly regulate drug release and pinpoint pharmacological activity. The poor absorption and bioavailability issues of phloretin have been solved by changing its dosage forms such as self-nanoemulsion (a 4- to 7-fold increase) [51], liposome [52], and microemulsion [53] formulation. In addition to this, researchers have successfully overcome the solubility obstacle of phloretin by synthesizing its derivatives [54,55]. Chemical synthesis processes would also satisfy the future demands of phloretin as it is present in little amount in organic matters. Several researchers have synthesized the analogs of the phloretin with improved biological activities and pharmacokinetic profile [54,55,56,57,58,59,60].

This review provides an outline of the plethora of research concerning the potential therapeutic benefits of phloretin along with major pharmacological targets and underlying molecular mechanisms. In addition, drug delivery approaches like nanotechnology and the development of analogs to overcome the poor bioavailability issues of phloretin are reviewed. We also reviewed extraction, purification, characterization, molecular structure, toxicity profile, as well as physicochemical, pharmacokinetic, and pharmaceutical characteristics of phloretin.

## 2. Extraction, Purification, and Characterization of Phloretin

**Figure 1:**
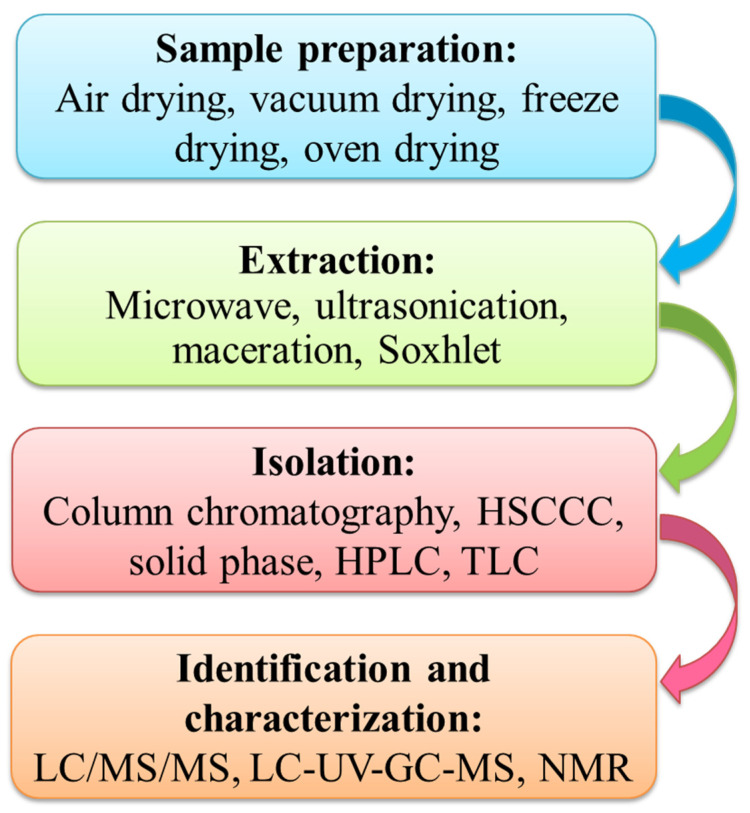
General scheme of sample preparation, extraction, isolation, identification, and characterization of phenolic compounds.

As phloretin is a phenolic compound, it is isolated by similar strategies to those routinely adopted for other phenolic compounds. Figure 1 represents a general scheme for sample preparation, extraction, isolation, identification, and characterization of phloretin (phenolics) [61]. Phloretin is extracted from various parts of the apple tree such as leaves, bark, fruits (flesh, pulp, pomace), and strawberries. It is isolated and characterized using various chromatographic techniques such as high-performance liquid chromatography (HPLC) and high-speed counter-current chromatography (HSCCC) coupled with modern spectroscopic techniques like mass spectrometry (MS) and nuclear magnetic resonance (NMR).

A brief procedure for extraction, isolation, and characterization of phloretin is described here as reported by Shim et al. [62]. Ground apples were homogenized in 70% methanol, and the resultant slurry was subjected to filtration. Subsequently, the apple extract was obtained by vacuum evaporation of the filtrate. The fractionation of extracts was carried out sequentially with n-hexane, chloroform, and ethyl acetate, yielding different fractions. The chloroform fraction was then exposed to silica gel column chromatography with an n-hexane and ethyl acetate gradient system to provide 10 fractions. Fraction 4 yielded a colorless crystal of phloretin. Furthermore, fraction 5 was extracted using semi-preparative HPLC to get crystals of the desired compound, which was subsequently identified as a phloretin by proton nuclear magnetic resonance (H-NMR) spectroscopy. Researchers extracted phloretin from various portions of the apple tree and strawberry fruits and subsequently performed characterization. The details are tabulated in Table 1 and Table 2.

## 3. Development of Analogs of Phloretin for the Improvement of Bioavailability

Phloretin is well-known for its various biological characteristics [54]. Despite several biological effects, the applications of phloretin in medications, food ingredients, and cosmetics have been restricted due to factors such as its low water solubility, low absorption, and low bioavailability [57,79]. Interestingly, by contrast, because of its better bioavailability and lower toxicity, phlorizin (phloridzin), a phloretin glycoside, is frequently utilized as a substitute for phloretin in numerous commercial products [50]. However, after oral administration, phlorizin is metabolized to phloretin and glucose by lactase hydrolase enzyme in the epithelial brush border membrane of the small intestine. The biological activities of phlorizin are thus a result of the activities of aglycone phloretin [44,45]. The presence of a little amount of phloretin in organic matter indicates the need to develop chemical synthesis processes that would satisfy future demands. As a result, it appears worthwhile to adopt a green approach to investigate chemical synthesis or hemi-synthesis to meet the demands of phloretin and its analogs in selected markets.

**Figure 2:**
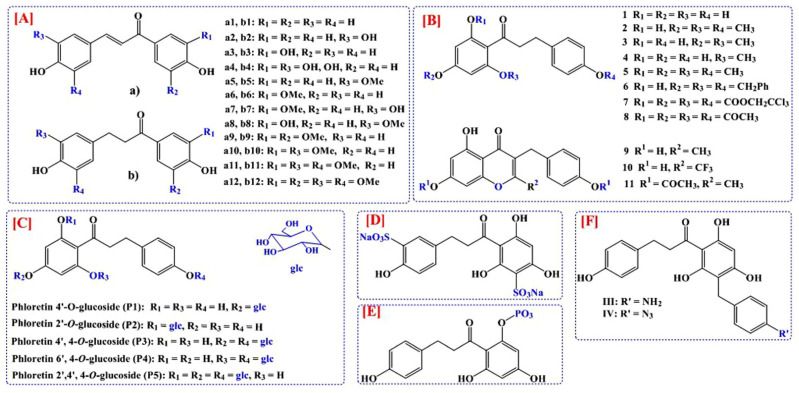
Some potential synthetic derivatives of phloretin: (**A**) Minsat et al. [56]; (**B**) Wang et al. [57]; (**C**) Pandey et al. [55]; (**D**) Shin et al. [54]; (**E**) Peerce et al. [59]; and (**F**) Diedrich [60].

Minsat et al. synthesized a library of 24 phloretin analogs (12 dihydrochalcones and 12 chalcones) (Figure 2A) using a Claisen–Schmidt condensation reaction as possible means for value addition in the cosmetic or nutraceutical industries [56]. Wang et al. synthesized a potential anticancer phloretin derivative (Figure 2B) [57]. A solubility obstacle of phloretin has been successfully overcome by researchers by synthesizing its derivatives [54,55]. By enhancing solubility, glycosylation improves the bioavailability and pharmacological characteristics of substances [58]. Pandey et al. demonstrated that the biosynthesis of varied glucosides of phloretin using glycosyltransferase (Figure 2C) enhances solubility and offers new biological effects [55]. Shin et al. synthesized water-soluble phloretin 3′,3-disulfonate (Figure 2D) (semi-synthetic phloretin) as a potential photoprotective agent [54]. Peerce et al. prepared a phosphorylated derivative of phloretin (Figure 2E) and evaluated Na^+^-dependent vesicular uptake of phosphate in the intestinal epithelial brush border [59]. P-aminobenzyl phloretin and P-azidobenzyl phloretin (Figure 2F) were synthesized by Diedrich as inhibitors of membrane glucose transport [60]. There is still scope for advancement in the production of additional semisynthetic/synthetic derivatives of phloretin which would help to overcome the issues such as poor water solubility, absorption and bioavailability, as well as in the quest for newer pharmacotherapeutic agents.

## 4. Pharmacokinetics of Phloretin

It is well established that the pharmacokinetics of a drug depends upon a variety of factors, including solubility, gastric pH, ionization, gastrointestinal stability, protein binding, and physiological factors [46]. The low water solubility of phloretin is responsible for its poor absorption and consequently lowers the bioavailability [47,48]. Crespy et al. observed that phloretin (aglycone) and phlorizin (glycone) were eliminated through urine when given to rats orally, and following 24 h of ingestion, 10.4% of the total amount was obtained from the urine. This study indicates the rapid absorption and elimination of phloretin [80]. Remsberg et al. monitored the pharmacokinetic profile of phloretin by HPLC methods. After intravenous administration of phloretin (10 mg/kg), the effects were observed for 6 h. Phloretin content quickly decreased within 30 min, and thereafter, a steady and complete elimination was noticed [49]. Moreover, Yuan Zhao et al. demonstrated the mechanism of absorption and bioavailability of phloretin in a single-pass intestinal perfusion using rats and in a Caco-2 cell monolayer. On oral administration, phloretin showed a poor bioavailability (8.67%), and the maximum absorption occurs from the colon through the mechanisms like cell bypass, efflux protein and active transportation. The efflux of phloretin via P-glycoprotein 1 (P-gp1) and multidrug resistance-associated protein 2 (MRP2) significantly lowers its intestinal transportation [46]. The poor absorption and bioavailability problem can be solved by changing its dosage forms such as self-nanoemulsions (a 4- to 7-fold increase) [51], liposome [52], and microemulsion [53] formulation. Also, co-administration of inhibitors of P-gp1 and MRP2 can enhance the bioavailability of phloretin.

## 5. Physicochemical and Pharmaceutical Characteristics of Phloretin

**Figure 3:**
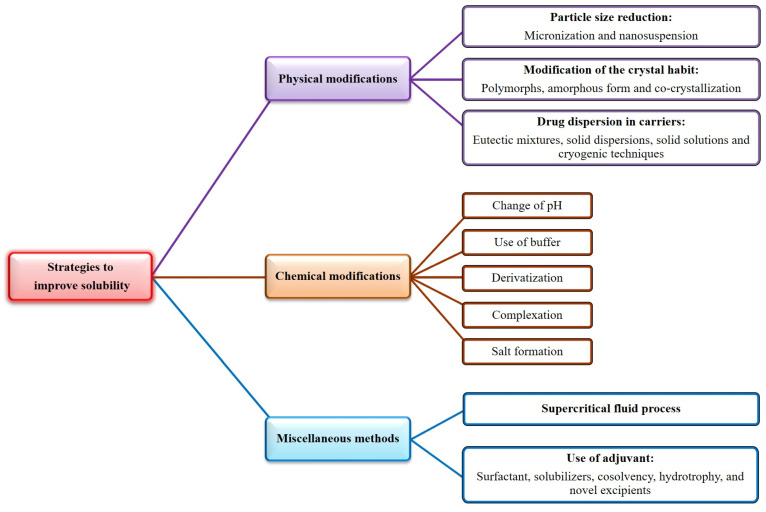
Various strategies to improve solubility.

Phloretin [3-(4-hydroxy phenyl)-1-(2′,4′,6′-trihydroxy phenyl)-propen-1-one] is a crystalline phenolic ketone. The major contributors to the various biological effects of phloretin are two aromatic phenol rings A and B, hydroxyl groups, and a carbonyl group. Moreover, 2, 6-dihydroxyacetophenone is considered as a vital pharmacophore responsible for the antioxidant effect of phloretin, wherein the involvement of carbonyl and OH groups of ring A increases the activity. However, the displacement of one hydroxyl moiety by a sugar (like in phlorizin) decreases the activity relative to phloretin [81]. The physiochemical characteristics of phloretin are tabulated in Table 2. Unfortunately, phloretin’s low solubility in water and lipids leads to its poor absorption and bioavailability [47,82,83]. Various ways used by researchers to increase drug solubility are depicted in Figure 3, which can be used to enhance the solubility of phloretin.

**Figure 4:**
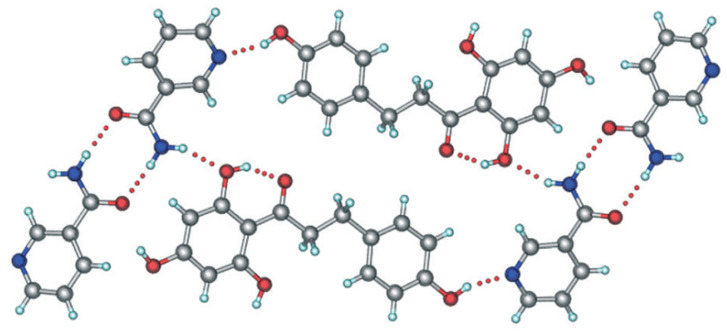
Packing diagram of the co-crystal of phloretin and nicotinamide (form I). Adapted with permission from Aitipamula et al. [85].

Co-crystals have recently acquired substantial momentum among physical modification approaches, owing to improving the physicochemical characteristics of active pharmaceutical ingredients (APIs) and the notion that their polymorphs behave in the same manner as that of pure APIs while exhibiting different attributes [84]. Aitipamula et al. synthesized novel polymorphs of a co-crystal of phloretin with nicotinamide. These polymorphs increased apparent solubility and dissolving rate, which was explained using characteristics derived from simulations of molecular dynamics [85]. The method adopted for co-crystallization of phloretin is briefly described here. In a stainless-steel grinding jar of 10 mL capacity, 200 mg of phloretin and 89 mg of nicotinamide were combined. Before grinding, to moisten the powder, two drops of solvent i.e., methanol was applied. The powder was ground at a 20 Hz rate with a 7 mm stainless steel ball for 20 min. Solvent-based co-crystallization investigations were carried out through dissolution of equimolar quantities of phloretin and nicotinamide in solvents such as methanol, ethanol, and 1,4-dioxane, and slow solvent evaporation at room temperature, yielding crystals of form I (Figure 4). The same study yielded phase pure form II when the co-crystallization solvent was a methanol-acetonitrile (1:1) combination. A rotovap technique was used to produce microcrystalline powders of form III. Powder X-ray diffraction was employed for identifying all crystalline powders. Before being used in future characterization and performance studies, the samples were sieved by Sonic Sifter Separator to ˂90 μm.

To further enhance the solubility, several researchers synthesized the derivatives of phloretin. The details are already discussed in the previous section, i.e., the development of analogs of phloretin. Similarly, to improve the absorption and bioavailability of phloretin, researchers developed various formulations such as self-nanoemulsion, nanoparticles, liposomes, and microemulsions. A thorough explanation of these approaches is elaborated in the following section, i.e., pharmaceutical development of phloretin.

## 6. Pharmacological Potentials and Molecular Mechanisms of Phloretin

Phloretin exerts various biological effects, and therefore, it may be beneficial for the treatment of several diseases as discussed below. A summary of therapeutic potential along with the possible mechanism of action of phloretin is given in Table 3.

### 6.1. Anticancer Activity

In the twenty-first century, cancer is one of the foremost reasons of mortality globally [86]. A plethora of research indicates phytochemicals as the emerging sources in the effective management of cancer [87]. In fact, several pieces of research have shown beneficial actions of phloretin against various kinds of cancer cells. It modulates a variety of cellular mechanisms involved in cancer including regulation of cellular growth, cell proliferation, apoptosis, angiogenesis, antioxidant defense mechanism, etc. [6,88].

In breast cancer cells such as estrogen-receptor-positive (MCF7) and triple-negative (MDA-MB-231) ones, phloretin downregulated the autophagy-related genes. It suppressed the expression of autophagosomal marker LC3B-II in low-glucose as well as in glucose-free media reflecting suppressed glucose-starvation-induced cytoprotective autophagy. Chemotherapy of ruthenium-phloretin complex promotes apoptosis in breast carcinoma cells by facilitating Bcl2 and Bax, and inhibiting PI3K/Akt/mTOR signaling [10]. It also attenuated doxorubicin- and tamoxifen-induced cytoprotective autophagy by downregulating mTOR/ULK1-triggered signaling mechanisms [3]. Phloretin shows beneficial effects on human oral cancer. It suppresses human SCC-1 oral cancer cell proliferation rate by reactive oxygen species (ROS) mediated apoptosis, and G0-G1 phase cell cycle arrest via reduction of cyclin D1, CDK4, and CDK6 expression [4]. Phloretin accelerates AGS gastric cancer cell death by G2/M phase arrest and inhibition of p-JNK and p38 expression [5]. Chemopreventive actions of phloretin are associated with the alteration of multiple signaling mechanisms. It arrests the cell cycle at the G0-G1 phase to inhibit the multiplication of human glioblastoma cells by enhancing the expression of p27 and reducing that of cdk2, cdk4, cdk6, cyclin D and cyclin E, and inhibiting the signaling of phosphatidylinositol 3 kinase/protein kinase B/mammalian target of rapamycin (PI3K/AKT/mTOR). It induces mitochondrial apoptosis mechanisms and augments ROS production along with the reduction in Bcl-2 and increase in Bax, Bak, and c-PARP [8]. In a recent study, a combination of phloretin with tumor necrosis factor-related apoptosis-inducing ligand (TRAIL) was evaluated in colon cancer. Phloretin potentiates the apoptosis triggered by TRAIL in HT-29-Luc cells [9]. Moreover, several reports indicate that phloretin possesses anticancer effects against several other cancer cells, including K562 leukemia cells, HL60 human leukemia cells, B16 melanoma, colon cancer cells, and hepatoma cell line via various cellular mechanisms such as arresting cell cycle, inducing apoptosis, activating of Bax and caspase expression, downregulating matrix metalloproteinase (MMP) expression, and reducing the occurrence of oxidative stress [6,7,88,89,90].

### 6.2. Antidiabetic Activity

Diabetes mellitus is a serious health issue worldwide that not only leads to serious consequences, but also shortens life expectancy. In the current scenario, phytomedicines and nutraceuticals have been shown to have promising effects on diabetes [91,92]. Several pieces of evidence point to the antidiabetic activity of phloretin [88]. Sodium-dependent glucose co-transporter 1 (SGLT1) in the apical brush border of intestinal epithelial cells and glucose transporter protein type 2 (GLUT2) in the basolateral intestinal membrane are involved in glucose absorption. Phloretin inhibits GLUT2 and thus reduces the basolateral exit of glucose from enterocytes to the blood [11,12]. Phloretin also inhibits SGLT1 and reduces the apical uptake of glucose. However, the inhibitory effect of phloretin on SGLT1 is significantly weaker compared to that of its glycone phlorizin (phloridzin) [11]. Interestingly, phlorizin is metabolized to phloretin and glucose by lactase hydrolase enzyme in the brush border enterocytes [11,93]. Therefore, administration of phlorizin via the oral route is likely to inhibit only SGLT1 and reduce the intestinal absorption of glucose; its other actions might be associated with the activities of the aglycone—phloretin [44]. In the kidney, phlorizin also inhibits SGLT2, responsible for renal tubular reabsorption of glucose, and thus increases the urinary excretion of glucose [13,14]. Viewed collectively, it seems that phloretin possesses the ability to lower blood glucose levels by inhibiting its intestinal absorption and increasing urinary excretion.

Recently, the antidiabetic potential of phloretin was reported against streptozotocin (STZ)-induced type 2 diabetes by regulating glucose and lipid metabolism. It activates the PI3K/AKT signaling cascade to enhance GLUT4 translocation and expression, resulting in improved glucose consumption and tolerance [15]. Phloretin not only attenuates the production of advanced glycation end products (AGEs) but also reduces receptor expression for AGEs by nuclear erythroid 2-related factor 2 (Nrf2)-dependent mechanisms to mitigate diabetes in mice induced by high-fat diet (HFD) [94]. In addition, a plethora of research indicates the antidiabetic potential of phloretin in animal models of diabetes. The studies suggest that phloretin improves the metabolism of glucose and lipid, sensitivity of insulin in peripheral tissues and antioxidant defense mechanism, and reduces the production of AGEs, expression of macrophage markers (F4/80 and Cd68), pro-inflammatory genes (MCP-1 and CCR2) and accumulation of lipid in adipose tissues [88,95,96]. Phloretin also seems to be effective in the management of diabetes-associated complications. Phloretin in a lower dose range preserves nephrin and podocin contents and exhibits a protective effect on podocytes in diabetic nephropathy by mechanisms other than those responsible for its hypoglycemic action [16]. Apple peel extract, a source of phloretin and phlorizin, was found effective in ameliorating diabetic peripheral neuropathy and augmenting wound healing in rats [34]. Phlorizin reversed the depression-like behavior in mice associated with diabetes [32]. Further, by reducing oxidative stress, inflammatory markers, and acetylcholine metabolism, and by increasing the neurotrophic factor, phlorizin improves memory deficits induced by diabetes or lipopolysaccharide (LPS) [33,35]. Hence, in the future, phloretin may emerge as an effective molecule for the management of diabetes and other metabolic disorders.

### 6.3. Antiobesity Activity

Type 2 diabetes is linked with overweight and obesity in about 80–85% of cases. Phytoconstituents are promising agents in the treatment of obesity [97,98]. Phloretin was found to inhibit adipogenicity by stimulating beta-catenin and apoptosis in adipocytes, at late stages of differentiation. Moreover, in adipocytes, phloretin stimulates osteoprotegerin (OPG) gene expression and OPG/RANKL ratio [99]. In the earlier study by Alsanea et al. [96], phloretin blocked weight gain induced by a high-fat diet in mice without causing weight loss. It also improved glucose homeostasis and insulin sensitivity, and reduced hepatic lipid accumulation. Phloretin reduces the expression of F4/80 and Cd68 (macrophage markers) and monocyte chemoattractant protein-1 and C-C motif chemokine receptor 2 (pro-inflammatory genes), and increases gene expression of adiponectin in white adipose tissue. Moreover, phloretin increases fatty acid oxidation gene expression such as carnitine palmitoyl transferase 1a and 1b, and reduces gene expression of de novo lipogenesis transcriptional factor peroxisome proliferator-activated receptor-γ 2 as well as its target monoacylglycerol O-acyltransferase. These pieces of evidence indicate the potential of phloretin for the mitigation of obesity and maintenance of metabolic homeostasis [96].

### 6.4. Cardiovascular Protective Activity

Currently, cardiovascular diseases (CVDs) like hypertension, atherosclerosis, stroke etc. are the foremost causes of death worldwide [100]. Daily consumption of healthy fruits and vegetables may reduce the risk of CVDs [101,102,103]. It is reported that phloretin inhibits platelets activation and tumor necrosis factor-alpha (TNF-α)-triggered expression of endothelial adhesion molecules in human umbilical vein endothelial cells (HUVECs) [17]. Some other studies revealed that phloretin shows protective effects against hydrogen peroxide-induced apoptosis in primary culture by inhibiting the chloride ion channels and hyperuricemia-induced endothelial dysfunction in HUVECs. In TNF-α-treated HUVECs, phloretin inhibits uric acid-triggered pro-inflammatory agents, nuclear factor-kappa B/extracellular signal-regulated kinase (NF-κB/p-ERK) contents and translocation of p65 subunit of NF-κB, and improves endothelial tube formation [18,19]. Phloretin protects the myocardium against doxorubicin-triggered injury in rats. Doxorubicin produces oxidative stress and decreases nitric oxide content in heart tissue, which is attenuated by phloretin. Treatment with phloretin also prevented doxorubicin-triggered changes in hemodynamic parameters (heart rate, mean arterial blood pressure, and left ventricular function) and decreased pro-inflammatory cytokine expression. The biochemical markers of myocardial injury like creatine kinase MB (CK-MB), lactate dehydrogenase (LDH), aspartate aminotransferase (AST), and alanine transaminase (ALT) were decreased by phloretin [104].

Diabetes significantly increases cardiovascular morbidity and mortality as the risk of myocardial infarction, stroke, and heart failure is much higher in diabetic patients [105]. Phloretin shows a protective effect on hyperglycemia-triggered injury in diabetic cardiomyopathy. This effect of phloretin was associated with the protection against inflammation injury on cardiomyocytes, and reduced fibrosis via restoring sirtuin 1 expression [20]. Diabetes doubles the risk of the development of hypertension. Hypertension coexists in more than 70% of the patients with diabetes, most likely due to underlying obesity [106]. Treatment with phlorizin decreased hyperglycemia by inhibiting SGLT2 in the kidney and consequently prevented the development of hypertension [14]. The findings suggest the effectiveness of phloretin in the management of cardiovascular comorbidities of diabetes.

### 6.5. Hepatoprotective Activity

It is well established that polyphenolic compounds show potential hepatoprotective activity. Recent studies demonstrated the protective effect of phloretin in experimental animals with liver damage induced by D-galactosamine, acetaminophen, and CCl4. Phloretin reduces the risk of liver damage by decreasing the contents of glutamic pyruvic transaminase (SGPT), serum glutamic-oxaloacetic transaminase (SGOT), gamma-glutamyl transferase (GGT), alkaline phosphatase (ALP), and total bilirubin in the serum. Moreover, phloretin alleviates oxidative stress and lipid peroxidation in liver tissue [21,22,23].

### 6.6. Anti-Inflammatory and Antioxidant Activities

Plants are the major sources of natural antioxidant and anti-inflammatory phytomedicines [107,108]. Phloretin displays these activities and, hence, can be used for the management of oxidative stress and inflammatory degenerative disease [26,109,110]. Phloretin shows significant antioxidant action against 2,2-diphenyl-1-picrylhydrazyl (DPPH; IC50 = 10 mmol/L) and 2,2′-azino-bis-3-ethylbenzothiazoline-6-sulfonic acid (ABTS; IC50 = 4.54 mmol/L) assays [29]. Also, phloretin decreases the matrix MMP-1, tyrosinase, and elastase activity. This antioxidant property of phloretin could be due to its dihydrochalcone structure [30,31]. Phlorizin (glycone of phloretin) ameliorates LPS-induced cognitive deficit, and diabetes-induced depression, memory impairment, delayed wound healing, and peripheral neuropathy by augmenting antioxidant defense mechanisms [32,33,34,35].

In the experimental autoimmune encephalomyelitis model, phloretin suppressed neuroinflammation via activation of Nrf2 signaling in macrophages, which occurs due to 5′ AMP-activated protein kinase (AMPK)-dependent activation of autophagy and consequent degradation of kelch-like ECH-associated protein 1 (Keap1) [24]. Phloretin mitigates inflammation in skin infection induced by *Propionibacterium acnes*. This effect was associated with inhibition of *Propionibacterium acnes*-triggered toll-like receptor (TLR) 2-mediated inflammatory pathway in the human keratinocytes [25]. Phloretin also reduced airway inflammation in asthmatic mice by alleviating the oxidative stress and infiltration of eosinophils. It alleviates the inflammation, antioxidant defense mechanism, lipid peroxidation, and cytokines formation by T helper 2 cells (Th2) in the bronchial tree, which validated its anti-inflammatory and anti-asthmatic activities [26]. Phloretin reduces the formation of pro-inflammatory cytokines like TNF-α, interleukin-6 (IL-6), IL-1, and IL-17 in mice with collagen-induced arthritis [27]. In dextran sulfate sodium-triggered ulcerative colitis in mice, phloretin improved histopathological changes in the colon. Moreover, it inhibited TNF-α, IL-1β, IL-12 IL-17A, and interferon-γ (IFN-γ) contents that were elevated due to ulcerative colitis. There was a significant activation of the NF-κB pathway, increased TLR4 expression, and reduced peroxisome proliferator-activated receptor-γ (PPARγ) expression in ulcerative colitis. These changes were restored by the phloretin. Escherichia coli and Lactobacillus levels were also re-balanced by the phloretin [2]. Hyperuricemia in mice induced renal dysfunction, which is associated with mitochondrial stress, inflammation, and renal fibrosis. Administration of phloretin decreased blood urea nitrogen (BUN) in serum, albumin to creatinine ratio (UACR) in urine, necrosis of tubules, deposition of extracellular matrix (ECM), and fibroblasts in the intestine. Moreover, it brought about a reduction in inflammatory cell infiltration, cytokines such as the NOD-like receptor family pyrin domain-containing 3 (NLRP3) and IL-1β, ROS in mitochondria and morphological lesions. Phloretin also increased urinary uric acid excretion partly due to inhibition of renal GLUT9. Phloretin also inhibited the NLPR3 pathway triggered by LPS or uric acid in HK-2 cells [111].

The molecular mechanisms behind the anti-inflammatory activities of phloretin are to inhibit the gene expression of pro-inflammatory agents, NF-κB, and mitogen-activated protein kinase (MAPK) pathway. In fact, phloretin reduces the formation of pro-inflammatory cytokines, such as IL-10, IL-8, IL-6, and TNF-α. Additionally, it also inhibits the inducible nitric oxide synthetase (iNOS) and cyclooxygenase-2 (COX2) activity and thereby reduces the formation of nitric oxide and prostaglandins [110,112,113]. Phloretin activates Nrf2 signaling to decrease the release of IL-8 triggered by LPS and thereby produces an anti-inflammatory effect in retinal pigment epithelium, and also inhibits the cellular glucose uptake [28]. Since satisfactory therapies for neuroinflammatory/neurodegenerative illnesses such as Alzheimer’s disease and Parkinsonism are not available [114,115], phloretin can be utilized as a promising molecule for these disorders.

### 6.7. Neuroprotective Activity

Alzheimer’s disease and Parkinson’s disease are the most common neurodegenerative diseases affecting millions of people worldwide. Although some drugs are available, they fail to halt the progressive neurodegeneration, and only temporarily improve the quality of life of the patient. Therefore, the identification of novel therapeutic agents particularly herbal-based medicine is essential. Some studies have reported the effectiveness of phloretin in these neurodegenerative diseases. Alzheimer’s disease is associated with extensive destruction of cholinergic neurons, neuroinflammation, oxidative stress, impairment of neuroplasticity, and deficits in learning and memory [114,115,116,117,118,119]. Phloretin treatment ameliorates scopolamine-induced amnesia in mice indicated by improved learning and memory performance of animals in the Morris water maze. In scopolamine-treated animals, it augments central cholinergic activity by inhibiting the activity of acetylcholinesterase. Moreover, the activities of antioxidant enzymes including reduced glutathione (GSH), superoxide dismutase (SOD) and catalase (CAT) were enhanced by phloretin. On the other hand, it decreases levels of lipid peroxidation product malonaldehyde (MDA), a biomarker of oxidative stress. Phloretin also promotes the neurotrophic activity that is indicated by elevated brain-derived neurotrophic factor (BDNF) levels in the hippocampus. These effects of phloretin were better than the centrally acting cholinesterase inhibitor donepezil, which is a clinically approved drug for Alzheimer’s disease [120]. In another experiment, Alzheimer’s disease was induced by intracerebroventricular administration of amyloid-β25-35 peptide in rats. Treatment with phloretin in these animals increased the spatial memory formation and retention in the Barnes maze test. Moreover, it decreases oxidative stress and TNF-α-triggered neuroinflammation. In the sections of the hippocampus stained with Congo red and Nissl stain, phloretin reduces the accumulation of amyloid-β in the cornu ammonis 1 (CA1) region and the number of pyknotic nuclei (indicators of degeneration) in the dentate gyrus (DG) [121]. Alzheimer’s disease induced by amyloid β1-42 peptide in rats showed a decrease in the levels of presynaptic protein synaptophysin (a marker of synaptic plasticity and integrity), Ki-67 (a nuclear protein associated with cellular proliferation) and doublecortin (a neuronal migration protein) in the brain. Phloretin pre-treatment exhibited a protective effect on the synaptophysin when measured by Western blotting. The immunohistochemistry data revealed that phloretin increased the count of Ki67- and doublecortin-containing cells in the DG. However, levels of postsynaptic density protein 95 (PSD-95) did not alter by phloretin [122].

Neurotoxicant MPTP administration in mice induced a Parkinson’s disease-like condition. In addition to motor abnormalities, it decreased dopamine levels and expression of tyrosine hydroxylase enzyme (the rate-limiting enzyme of dopamine biosynthesis). Moreover, indicators of neuroinflammation such as astrocytes marker glial fibrillary acidic protein (GFAP), microglial marker ionized calcium-binding adaptor protein-1 (iba1), iNOS and COX2 were overexpressed in MPTP intoxicated animals. In addition to this, the levels of proinflammatory cytokines including IL-β, IL-6, and TNF-α were significantly increased by MPTP. All these changes were effectively restored by treatment with phloretin [123]. Viewed collectively, the above data suggest that phloretin improves the hallmark neuropathological features of Alzheimer’s disease and Parkinson’s disease.

### 6.8. Immunosuppressant Activity

Lu et al. have reported the immunosuppressive activity of phloretin in mice using carboxyfluorescein diacetate succinimidyl ester staining plus flow cytometry assay. They proposed that phloretin suppresses the proliferation of T lymphocytes and also inhibits the expression of cluster of differentiation (CD69, CD25), and causes cell G0/G1 phase cycle arrest [36].

### 6.9. Antimicrobial Activity

Natural resources can be explored to discover effective antimicrobial molecules with a high degree of safety. Significant pieces of evidence suggest that dietary polyphenols may possess an excellent antimicrobial activity. Although the mechanism of action is unclear, phloretin was found to be effective against various pathogenic microorganisms. Phloretin inhibits gram-positive bacteria, especially *Staphylococcus aureus*, and methicillin-resistant *Staphylococcus aureus* clinical strains [39]. Furthermore, phloretin considerably decreases the *Salmonella typhimurium* bacterial load of infected mice [42]. Some reports showed that phloretin is also effective against *Listeria monocytogenes* [40,41]. In rats, phloretin suppresses the production of *Escherichia coli* O157:H7 biofilm and reduces inflammatory reactions in the colon without causing harm to the beneficial commensal *Escherichia coli* biofilms [37]. In a mice model of oral candidiasis, phloretin was found to be effective against *Candida albicans* infection without inducing any tissue necrosis [124]. Moreover, an in vitro study showed the effectiveness of phloretin against a number of plant pathogenic fungi *Phytophthora capsici*, *Alternaria panax*, *Sclerotinia sclerotiorum*, *Rhizoctonia solani* AG4, and *Magnaporthe grisea* on rice and tomato seedlings [62]. These findings indicate the potential of phloretin to develop into a natural preservative in the food industry [41].

It is well established that *Mycobacterium tuberculosis* infection leads to severe lung inflammation and it has been reported that phloretin decreases the expression of inflammatory agents such as IL and TNF-α to show antimicrobial activity. Furthermore, RT-PCR and immunoblot analysis for the evaluation of the action of phloretin on interferon-stimulated MRC-5 human lung fibroblasts and LPS-stimulated dendritic cells suggest the inhibition of the secretion of IL-1, IL-12, and TNF-α, the mRNA expression of IL-1, IL-6, IL-12, TNF-α and MMP-1, and the phosphorylation of p38 MAPK and ERK [38].

**Table 3:** Therapeutic potential and molecular mechanisms of phloretin.

SN	Activity	Mechanism	Reference
1.	Anticancer		
	a. Triple-negative MDA-MB-231 and estrogen-receptor-positive MCF7 breast cancer cells	Reduced LC3B-II expression in low-glucose and glucose-free media; Reversed doxorubicin- and tamoxifen-induced cytoprotective autophagy; Downregulated mTOR/ULK1 signaling; Facilitated apoptosis through Bcl2 and Bax; Downregulated PI3K/Akt/mTOR signaling	[3,10]
	b. SCC-1 oral cancer cells	ROS mediated cell death; Arrested cell cycle in G0-G1 phase; Reduced expression of cyclin D1, CDK4, and CDK6	[4]
	c. AGS gastric cancer cells	Arrested cell cycle in G2/M phase; Reduced p-JNK and p38 expression	[5]
	d. Human glioblastoma cells	Arrested cell cycle in G0-G1 phase; Increased p27 expression; Decreased cdk2, cdk4, cdk6, cyclin D, and cyclin E expressions; Inhibited PI3K/AKT/mTOR signaling; Induced mitochondrial apoptosis pathways; Increased ROS production; Down-regulated Bcl-2; Up-regulated Bax, Bak, and c-PARP	[8]
	e. Esophageal squamous cell carcinoma cells, EC109	Increased activity of p53; Increased levels of Bax; Decreased levels of Bcl-2	[6,125]
	f. Non-small cell lung cancer: A549, Calu-1, H838, and H520 cells	Decreased Bcl-2 expression; Increased cleaved-caspase-3 and -9 protein expression; Deregulated MMP-2 and -9 gene expression and protein levels	[6,7]
	g. Human erythroid leukemia cells, K-562	Increased HSP70 penetration efficacy; Potentiated antitumor activity of HSP70	[6,126]
	h. HepG2-xenografted tumor	Potentiated anticancer effect of paclitaxel	[6,127]
	i. HepG2 human ileocecal cancer cell line, HT-29 human colon cancer cell line, Bel 7402 liver cancer cell line, and A549 human lung cancer cell line	Marked anticancer activity	[6,9,64]
2.	Antidiabetic	Inhibited intestinal SGLT1 and GLUT2 to reduce glucose absorption	[11,12]
		Inhibited renal SGLT2 to reduce renal tubular reabsorption of glucose, and thus increased urinary excretion of glucose	[13,14]
		Activated PI3K/AKT signaling cascade by GLUT4 translocation and expression to improve glucose consumption and tolerance in type 2 diabetes	[15]
		Inhibited production of AGEs and suppressed receptor expression for AGEs by Nrf2-dependant pathway and mitigated HFD-induced diabetes in C57BL/6 mice	[94]
		Preserved nephrin and podocin contents to protect podocytes in diabetic nephropathy	[16]
3.	Antiobesity	Inhibited adipogenicity by stimulating beta-catenin and adipocytes apoptosis; Stimulated phloretin OPG gene expression and OPG/RANKL ratio in adipocytes	[99]
		Blocked weight gain induced by high-fat diet feeding; Reduced hepatic lipid accumulation; Reduced expression of macrophage markers and pro-inflammatory genes and increased adiponectin gene in white adipose tissue; Increased fatty acid oxidation genes expression and reduced expression of lipogenesis transcriptional factor	[96]
4.	Cardioprotective	Reduced the activation of platelets and TNF-induced expression of endothelial adhesion molecules in HUVECs	[17]
		Protected against hydrogen peroxide-induced apoptosis in primary culture by inhibiting the chloride ion channels; Inhibited uric acid-induced pro-inflammatory factors, p-NF-κB/p-ERK levels, and nuclear translocation of NF-κΒ p65, and improved endothelial tube formation in TNF-treated HUVECs	[18,19]
		Protected myocardium against doxorubicin-triggered injury; Attenuated doxorubicin-produced oxidative stress and decreased nitric oxide contents in heart tissue; Prevented doxorubicin-triggered changes in hemodynamic parameters; Decreased pro-inflammatory cytokines, and plasma myocardial injury markers such as CK-MB, LDH, AST and ALT	[104]
		Protected against hyperglycemia-triggered injury in diabetic cardiomyopathy by reducing fibrosis via restoring sirtuin 1 expression	[20]
		Decreased hyperglycemia by inhibiting SGLT2 in the kidney and consequently prevented the development of hypertension	[14]
5.	Hepatoprotective	Protected against acetaminophen, CCl_4,_ and D-galactosamine-induced acute liver damage; Decreased levels of ALT, AST, GGT, ALP, and total bilirubin levels; Alleviated oxidative stress and lipid peroxidation in liver tissue	[21,22,23]
6.	Anti-inflammatory	Activated Nrf2 signaling to decrease the release of IL-8 triggered by LPS and thereby produced anti-inflammatory effect in retinal pigment epithelium (ARPE-19 cells); Inhibited the glucose uptake in ARPE-19 cells	[28]
		Suppressed neuroinflammation in experimental autoimmune encephalomyelitis model via activation of Nrf2 signaling in macrophages, attributed to AMPK-dependent activation of autophagy and consequent degradation of Keap1	[24]
		Reduced levels of BUN, UACR, tubular necrosis, ECM deposition, and interstitial fibroblasts in mice with hyperuricemia-induced renal dysfunction; Reduced renal inflammatory cells infiltration, cytokines (NLRP3 and IL-1β), mitochondrial ROS, and morphological lesions; Inhibited renal GLUT9 and promoted urinary uric acid excretion	[111]
		Inhibited *Propionibacterium acnes*-induced TLR2-mediated inflammatory signaling in human keratinocytes	[25]
		Improved histopathological changes in the colon of mice with dextran sulfate sodium-induced ulcerative colitis; Inhibited TNF-α, IL-1β, IL-12 IL-17A and IFN-γ levels; Activation of NF-κB pathway, increased TLR4 expression, and reduced PPARγ expression in ulcerative colitis were restored; *Escherichia coli* and *Lactobacillus* levels were re-balanced	[2]
		Reduced inflammation, eosinophil infiltration, Th2 cytokine production, and oxidative stress in ovalbumin-induced asthmatic mice	[26]
		Reduced formation of inflammatory cytokines (TNF, IL-6, IL-1, and IL-17) in collagen-induced arthritic mice	[27]
7.	Antioxidant	Antioxidant action in DPPH and ABTS assay	[29]
		Reduced the matrix MMP-1, tyrosinase, and elastase activity due to its dihydrochalcone structure	[30,31]
		Augmented antioxidant defense mechanisms by phlorizin (glycone of phloretin) to ameliorate LPS-induced cognitive deficit, and diabetes-induced depression, memory impairment, delayed wound healing, and peripheral neuropathy	[32,33,34,35]
8.	Neuroprotective		
	a. Alzheimer’s disease	Improved learning and memory performance of animals with Alzheimer’s disease-like condition induced by scopolamine and amyloid β; Inhibited activity of acetylcholinesterase; Increased BDNF levels;Decreased oxidative stress by enhancing activities of GSH, SOD and CAT, and decreasing levels of MDA; Decreased TNF-α-triggered neuroinflammation; Reduced accumulation of amyloid-β in the CA1 hippocampal region and number of pyknotic nuclei in the hippocampal DG;Exhibited protective effect on the synaptophysin; Increased count of Ki67- and doublecortin in the DG; No change in PSD-95 levels	[120,121,122]
	b. Parkinson’s disease	Improved motor performance of animals with Parkinson’s disease-like condition induced by MPTP; Increased dopamine levels and tyrosine hydroxylase enzyme expression; Suppressed neuroinflammation: reduced expression of GFAP, iba1, iNOS and COX2; Reduced levels of proinflammatory cytokines (IL-β, IL-6, and TNF-α)	[123]
9.	Immunosuppressant	Suppressed proliferation of T lymphocytes and expression of CD69 and CD25, and arrested cell cycle in G0/G1 phase	[36]
10.	Antimicrobial	Suppressed production of *Escherichia coli* O157:H7 biofilm and reduced colon inflammation without causing harm to the beneficial commensal *Escherichia coli* biofilms	[37]
		Inhibited *Mycobacterium tuberculosis* by reducing the expression of inflammatory molecules such as ILs and TNF-α	[38]
		Inhibited *Staphylococcus aureus*, *Listeria monocytogenes*, methicillin-resistant *Staphylococcus aureus* clinical strains, and *Salmonella typhimurium*	[39]
		Inhibited *Listeria monocytogenes*	[40,41]
		Decreased *Salmonella typhimurium* bacterial load of infected mice	[42]
		Inhibited *Candida albicans* without inducing any tissue necrosis in mouse model of oral candidiasis	[124]
		Inhibited plant pathogenic fungi *Phytophthora capsici*, *Alternaria panax*, *Sclerotinia sclerotiorum*, *Rhizoctonia solani* AG4, and *Magnaporthe grisea* on rice and tomato seedlings	[62]

## 7. Safety and Adverse Effects of Phloretin

Extensive research is required for the exploration of the adverse effects of phloretin. A study by Geohagen et al. found the dose-dependent toxicity of phloretin. The highest intraperitoneal dose of phloretin (2.4 mmol/kg) in mice showed 100% lethality. Oral administration of phloretin at the highest dose (2.4 mmol/kg) caused 64% mortality. At a lower dose range, phloretin was safe [43]. Phlorizin (phloridzin), a glucoside of phloretin, was also evaluated for its effect on the musculoskeletal system. In HFD-STZ-induced diabetic rats, phlorizin was orally administered for 4 weeks. The diabetic animals showed a significant decrease in muscle mass/strength, and marked osteoporotic changes. These unfavorable outcomes of diabetes were significantly augmented by a lower dose of phlorizin (20 mg/kg). Interestingly, at a higher dose (50 mg/kg), musculoskeletal parameters were not affected significantly. This might be associated with the antioxidant effect of phlorizin at a higher dose [44].

## 8. Pharmaceutical Development of Phloretin

**Figure 5:**
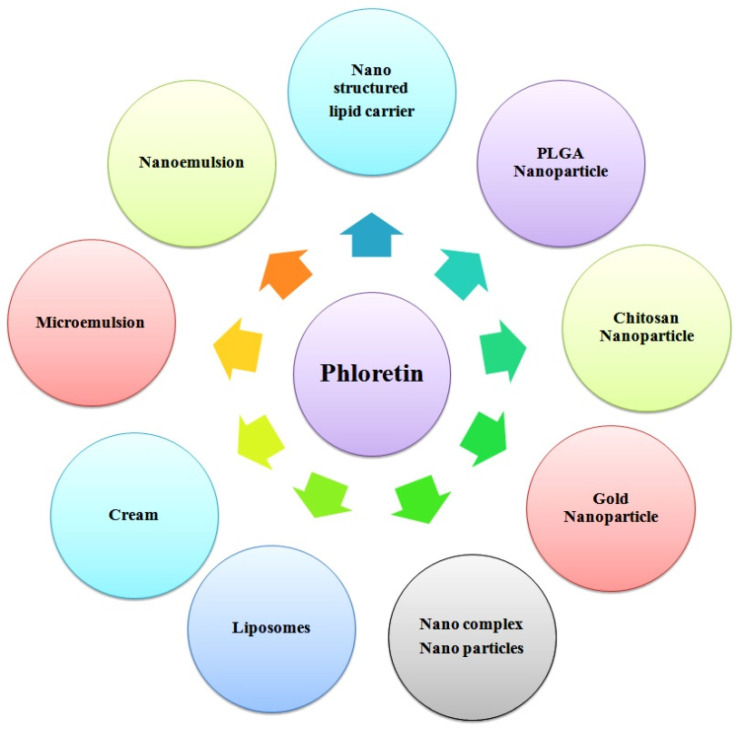
Drug delivery systems to improve absorption and bioavailability of phloretin.

Many strategies have been found in recent years to design formulations with enhanced pharmacokinetic qualities to gain optimum therapeutic benefits of phytoconstituents and other molecules [128,129,130,131,132,133]. Unfortunately, the limited solubility of phloretin in water and lipids results in poor absorption and bioavailability, limiting its usage in traditional drug delivery systems. To overcome the disadvantages of phloretin, researchers devised novel drug delivery systems to improve absorption and bioavailability (Figure 5). Among these, nanotechnology is one of the potential techniques to overcome the aforementioned challenges by significantly regulating drug release and pinpointing pharmacological activity. Nanoformulation-based techniques can improve the dissolution of poorly soluble pharmaceuticals, increasing their stability and bioavailability, and decreasing their toxicity. Nanocarriers come in a variety of forms, including nanoemulsion, nanostructured lipid carriers, liposomes, polymeric nanoparticles, gold nanoparticles, and solid lipid nanoparticles.

Chen et al. prepared a ternary nanocomplex of carboxymethyl phytoglycogen, caseinate, and pectin as a carrier for the oral delivery of phloretin [134]. To enhance the bioavailability and cellular uptake of phloretin, pH-sensitive chitosan nanoparticles were formulated and evaluated by Mariadoss et al. [135]. Gu et al. fabricated the nanostructured lipid carrier for enhancing the absorption of phloretin [136]. Similarly, Casarini et al. encapsulated phloretin in nanocapsules to enhance its water solubility. Further, hydrogel-containing nanocapsules were fabricated for targeting melanoma [137]. Similarly, Nam et al. prepared phloretin containing fast dissolving nanofibers for targeting squamous cell cancer [138].

A phloretin nanostructured lipid carrier was successfully synthesized and evaluated for potential antioxidant activity [136]. Similarly, Ranjanamala et al. evaluated the dose-dependent antioxidant and antimicrobial properties of phloretin encapsulated PLGA nanoparticles [139]. Payne et al. also designed and evaluated gold nanoparticles of phloretin for antineoplastic activity [140]. Auner et al. investigated the penetration-enhancing properties of phloretin in liposomes in conjunction with 6-ketocholestanol. The increased fluidity of the intercellular lipid bilayers of the stratum corneum is thought to be responsible for the penetration-enhancing action of phloretin and 6-ketocholestanol [30]. Guo et al. [47] and Yang et al. [141] have also created a liposomal formulation for phloretin.

Wang et al. created a polymeric self nanoemulsion to improve the bioavailability and bioefficacy of phloretin. The results revealed that nanoemulsion improves bioavailability by 7.9 times and anti-inflammatory effect by 6.8 times over plain phloretin [51]. Simultaneously, Abu-Azzam and Nasr investigated the anti-inflammatory activity of a microemulsion formulation of phloretin for vaginal irritation [53]. Kum et al. [142] and Pinnell et al. [143] evaluated the anti-acne and anti-aging activities of phloretin-containing cream. Furthermore, Hu et al. produced a phloretin inclusion complex to improve its water solubility and antioxidative potential [144].

## 9. Conclusion and Future Directions

The present review enumerates the biological actions of phloretin and reveals its therapeutic potential in various conditions like cancers, diabetes, liver damage, kidney damage, encephalomyelitis, ulcerative colitis, asthma, arthritis, and cognitive impairment. By virtue of low toxicity, phloretin and its glycone phlorizin may gain acceptance for pharmacological research and subsequent development for clinical uses. The multifunctional and poly-pharmacological approaches can provide a rationale for the application of phloretin-based therapy in diseases involving multifactorial pathogenesis. Further, its combination therapy with conventional drugs may enhance the effectiveness of the therapy while lessening their adverse effects. Despite available data, we feel that further investigations on phloretin are required to be carried out. The precise molecular mechanisms by which phloretin exhibits its biological actions still need further elucidations. There is still a huge scope in evaluating the effects of phloretin in neuropsychiatric- and metabolic disorders. To enhance water solubility and bioavailability, more synthetic/semisynthetic derivatives of phloretin must be developed. Nanoformulations can also be developed further to enhance bioavailability. Furthermore, the structure-activity relationships require to be investigated for the development of druggable congeners. Being a natural constituent present in apples and strawberries, phloretin therapy seems to be safe. However, one study shows its toxicity in mice at a high dose. Therefore, additional safety, as well as regulatory toxicity studies are required. Taken together, the biological and pharmacological actions, molecular mechanisms and therapeutic potentials of phloretin make it a promising candidate for drug development.

## Figures and Tables

**Table 1:** Extraction and characterization of phloretin.

Plant Part	Extraction Method	Extraction Solvent	Analytical Technique	Reference
Apple fruits	Solvent extraction	Methanol, n-hexane, CHCl_3,_ ethyl acetate	HPLC-NMR	[62]
Apple leaves	Homogenization, centrifugation	Methanol, acetone	HPLC	[63]
Apple leaves	Solvent extraction	Ethanol, water	HPLC	[64]
Apple leaves	Ultrasound	Ethanol, water	LCMS	[65]
Apple leaves, bark, and buds	Centrifugation and sonication	Methanol, formic acid	HPLC-DAD	[66]
Apple peel, flesh, and leaves	Solvent extraction	Methanol, water	UPLC-DAD-HESI-MS	[67]
Apple pomace	Solvent extraction	Acetone, methanol, ethanol, ethyl acetate	RP-HPLC-DAD	[68]
Apple pomace	Solvent extraction	Acetone, methanol, ethanol, CHCl_3_, ethyl acetate	HPLC-DAD	[69]
Apple pulp and peel	Solvent extraction	Methanol	HPLC-NMR-MS	[70]
Apple pulp and peel	Solvent extraction and sonication	Methanol (1% HCl)	HPLC	[71]
Apple tree bark	Solvent extraction	Ethanol, ethyl acetate	HSCCC	[72]
Strawberry fruits	Homogenization, solvent extraction	Acetone, ethyl acetate, methanol	HPLC−PDA−MS/MS and NMR	[73]
Strawberry fruits	Centrifugation, solvent extraction	Methanol (HCl:Water, 50:50)	UPLC−PDA−MS/MS and NMR	[74]
Strawberry fruits	Solvent extraction	Acetone, water	UPLC−MS/MS	[75]
Strawberry pomace	Solvent extraction	Water, ethanol	HPLC-DAD	[76]

**Table 2:** Molecular structure and other physiochemical details of phloretin.

Particulars	Data	Reference
Molecular structure and other details	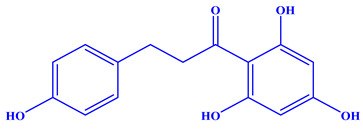	[77]
	Molecular formula: C_15_H_14_O_5_	
	Synonyms: dihydronaringenin, phloretol	
	Color: pearl white powder	
	Melting point: 263.5 °C	
	Molecular weight: 274.27	
	Solubility: slightly soluble in water, sparingly soluble in methanol, ethanol, propan-1-ol, propan-2-ol, butan-1-ol, butan-2-ol, pentan-1-ol, hexan-1-ol, ethyl acetate, butyl acetate, and 1,4-dioxane and DMSO	[78]
Spectroscopic analysis	UV-Visible: λ = 225, 282.8, 369	[67,68]
	^1^H NMR: δ: 2.86 (2H, t, *J* = 7.6 Hz, H-β), 3.20 (2H, t, *J* = 7.6 Hz, H-α), 5.80 (2H, s, H-3′, 5′), 6.65 (2H, d, *J* = 8.5 Hz, H-3, 5), 7.00 (2H, d, *J* = 8.5 Hz, H-2,6)	[62,64]
	^13^C NMR: 132.6, 128.93, 114.7, 155.03, 114.7, 128.93, 103.91, 164.74, 94.34, 164.44, 94.34, 164.74, 45.93, 30.09, 205	[64]

## Data Availability

Not applicable.

## References

[1] Roy A., Khan A., Ahmad I., Alghamdi S., Rajab B.S., Babalghith A.O., Alshahrani M.Y., Islam S., Islam M. (2022). Flavonoids a Bioactive Compound from Medicinal Plants and Its Therapeutic Applications. Biomed. Res. Int..

[2] Zhang Z., Li S., Cao H., Shen P., Liu J., Fu Y., Cao Y., Zhang N. (2019). The protective role of phloretin against dextran sulfate sodium-induced ulcerative colitis in mice. Food Funct..

[3] Chen M., Gowd V., Wang M., Chen F., Cheng K.-W. (2021). The apple dihydrochalcone phloretin suppresses growth and improves chemosensitivity of breast cancer cells via inhibition of cytoprotective autophagy. Food Funct..

[4] Yang G., Yin X., Ma D., Su Z. (2020). Anticancer activity of Phloretin against the human oral cancer cells is due to G0/G1 cell cycle arrest and ROS mediated cell death. J. BUON.

[5] Xu M., Gu W., Shen Z., Wang F. (2018). Anticancer Activity of Phloretin Against Human Gastric Cancer Cell Lines Involves Apoptosis, Cell Cycle Arrest, and Inhibition of Cell Invasion and JNK Signalling Pathway. Med. Sci. Monit..

[6] Choi B.Y. (2019). Biochemical basis of anti-cancer-effects of phloretin—A natural dihydrochalcone. Molecules.

[7] Ma L., Wang R., Nan Y., Li W., Wang Q., Jin F. (2016). Phloretin exhibits an anticancer effect and enhances the anticancer ability of cisplatin on non-small cell lung cancer cell lines by regulating expression of apoptotic pathways and matrix metalloproteinases. Int. J. Oncol..

[8] Liu Y., Fan C., Pu L., Wei C., Jin H., Teng Y., Zhao M., Yu A.C.H., Jiang F., Shu J. (2016). Phloretin induces cell cycle arrest and apoptosis of human glioblastoma cells through the generation of reactive oxygen species. J. Neurooncol..

[9] Kim J.L., Lee D.H., Pan C.H., Park S.J., Oh S.C., Lee S.Y. (2022). Role of phloretin as a sensitizer to TRAIL-induced apoptosis in colon cancer. Oncol. Lett..

[10] Roy S., Mondru A.K., Chakraborty T., Das A., Dasgupta S. (2022). Apple polyphenol phloretin complexed with ruthenium is capable of reprogramming the breast cancer microenvironment through modulation of PI3K/Akt/mTOR/VEGF pathways. Toxicol. Appl. Pharmacol..

[11] Schulze C., Bangert A., Kottra G., Geillinger K.E., Schwanck B., Vollert H., Blaschek W., Daniel H. (2014). Inhibition of the intestinal sodium-coupled glucose transporter 1 (SGLT1) by extracts and polyphenols from apple reduces postprandial blood glucose levels in mice and humans. Mol. Nutr. Food Res..

[12] Kellett G.L., Helliwell P.A. (2000). The diffusive component of intestinal glucose absorption is mediated by the glucose-induced recruitment of GLUT2 to the brush-border membrane. Biochem. J..

[13] Tahrani A.A., Barnett A.H., Bailey C.J. (2013). SGLT inhibitors in management of diabetes. Lancet Diabetes Endocrinol..

[14] Osorio H., Bautista R., Rios A., Franco M., Arellano A., Vargas-Robles H., Romo E., Escalante B. (2010). Effect of phlorizin on SGLT2 expression in the kidney of diabetic rats. J. Nephrol..

[15] Shen X., Wang L., Zhou N., Gai S., Liu X., Zhang S. (2020). Beneficial effects of combination therapy of phloretin and metformin in streptozotocin-induced diabetic rats and improved insulin sensitivity in vitro. Food Funct..

[16] Liu J., Sun M., Xia Y., Cui X., Jiang J. (2022). Phloretin ameliorates diabetic nephropathy by inhibiting nephrin and podocin reduction through a non-hypoglycemic effect. Food Funct..

[17] Stangl V., Lorenz M., Ludwig A., Grimbo N., Guether C., Sanad W., Ziemer S., Martus P., Baumann G., Stangl K. (2005). The flavonoid phloretin suppresses stimulated expression of endothelial adhesion molecules and reduces activation of human platelets. J. Nutr..

[18] Malekova L., Tomaskova J., Novakova M., Stefanik P., Kopacek J., Lakatos B., Pastorekova S., Krizanova O., Breier A., Ondrias K. (2007). Inhibitory effect of DIDS, NPPB, and phloretin on intracellular chloride channels. Pflugers Arch..

[19] Liu S., Yuan Y., Zhou Y., Zhao M., Chen Y., Cheng J., Lu Y., Liu J. (2017). Phloretin attenuates hyperuricemia-induced endothelial dysfunction through co-inhibiting inflammation and GLUT9-mediated uric acid uptake. J. Cell. Mol. Med..

[20] Ying Y., Jiang C., Zhang M., Jin J., Ge S., Wang X. (2019). Phloretin protects against cardiac damage and remodeling via restoring SIRT1 and anti-inflammatory effects in the streptozotocin-induced diabetic mouse model. Aging.

[21] Ebadollahi Natanzi A.R., Mahmoudian S., Minaeie B., Sabzevari O. (2011). Hepatoprotective activity of phloretin and hydroxychalcones against Acetaminophen Induced hepatotoxicity in mice. Iran. J. Pharm. Sci..

[22] Zuo A.R., Yu Y.Y., Shu Q.L., Zheng L.X., Wang X.M., Peng S.H., Xie Y.F., Cao S.W. (2014). Hepatoprotective effects and antioxidant, antityrosinase activities of phloretin and phloretin isonicotinyl hydrazone. J. Chin. Med. Assoc..

[23] Lu Y., Chen J., Ren D., Yang X., Zhao Y. (2017). Hepatoprotective effects of phloretin against CCl4-induced liver injury in mice. Food Agric. Immunol..

[24] Dierckx T., Haidar M., Grajchen E., Wouters E., Vanherle S., Loix M., Boeykens A., Bylemans D., Hardonnière K., Kerdine-Römer S. (2021). Phloretin suppresses neuroinflammation by autophagy-mediated Nrf2 activation in macrophages. J. Neuroinflamm..

[25] Cheon D., Kim J., Jeon D., Shin H.-C., Kim Y. (2019). Target Proteins of Phloretin for Its Anti-Inflammatory and Antibacterial Activities Against Propionibacterium acnes-Induced Skin Infection. Molecules.

[26] Huang W.C., Fang L.W., Liou C.J. (2017). Phloretin Attenuates Allergic Airway Inflammation and Oxidative Stress in Asthmatic Mice. Front. Immunol..

[27] Wang S.P., Lin S.C., Li S., Chao Y.H., Hwang G.Y., Lin C.C. (2016). Potent Antiarthritic Properties of Phloretin in Murine Collagen-Induced Arthritis. Evid. Based. Complement. Alternat. Med..

[28] Hytti M., Ruuth J., Kanerva I., Bhattarai N., Pedersen M.L., Nielsen C.U., Kauppinen A. (2022). Phloretin inhibits glucose transport and reduces inflammation in human retinal pigment epithelial cells. Mol. Cell. Biochem..

[29] Leu S.J., Lin Y.P., Lin R.D., Wen C.L., Cheng K.T., Hsu F.L., Lee M.H. (2006). Phenolic constituents of Malus doumeri var. formosana in the field of skin care. Biol. Pharm. Bull..

[30] Auner B.G., O’Neill M.A.A., Valenta C., Hadgraft J. (2005). Interaction of phloretin and 6-ketocholestanol with DPPC-liposomes as phospholipid model membranes. Int. J. Pharm..

[31] Nakamura Y., Watanabe S., Miyake N., Kohno H., Osawa T. (2003). Dihydrochalcones:  Evaluation as Novel Radical Scavenging Antioxidants. J. Agric. Food Chem..

[32] Kamdi S.P., Raval A., Nakhate K.T. (2021). Phloridzin ameliorates type 2 diabetes-induced depression in mice by mitigating oxidative stress and modulating brain-derived neurotrophic factor. J. Diabetes Metab. Disord..

[33] Kamdi S.P., Raval A., Nakhate K.T. (2021). Phloridzin attenuates lipopolysaccharide-induced cognitive impairment via antioxidant, anti-inflammatory and neuromodulatory activities. Cytokine.

[34] Kamdi S.P., Raval A., Nakhate K.T. (2021). Effect of apple peel extract on diabetes-induced peripheral neuropathy and wound injury. J. Diabetes Metab. Disord..

[35] Kamdi S.P., Badwaik H.R., Raval A., Ajazuddin, Nakhate K.T. (2021). Ameliorative potential of phloridzin in type 2 diabetes-induced memory deficits in rats. Eur. J. Pharmacol..

[36] Lu X., Zeng Y., Ye Y., Zhou Y., Mu J., Zhao X. (2009). Anti-inflammatory and immunosuppressive effect of phloretin. Yao Xue Xue Bao.

[37] Lee J.H., Regmi S.C., Kim J.A., Cho M.H., Yun H., Lee C.S., Lee J. (2011). Apple flavonoid phloretin inhibits *Escherichia coli* O157: H7 biofilm formation and ameliorates colon inflammation in rats. Infect. Immun..

[38] Jeon D., Jeong M.-C., Jnawali H.N., Kwak C., Ryoo S., Jung I.D., Kim Y. (2017). Phloretin Exerts Anti-Tuberculosis Activity and Suppresses Lung Inflammation. Molecules.

[39] Barreca D., Bellocco E., Laganà G., Ginestra G., Bisignano C. (2014). Biochemical and antimicrobial activity of phloretin and its glycosilated derivatives present in apple and kumquat. Food Chem..

[40] Wang J., Liu B., Teng Z., Zhou X., Wang X., Zhang B., Lu G., Niu X., Yang Y., Deng X. (2017). Phloretin Attenuates *Listeria monocytogenes* Virulence Both In vitro and In vivo by Simultaneously Targeting Listeriolysin O and Sortase A. Front. Cell. Infect. Microbiol..

[41] Zhao P., Zhang Y., Deng H., Meng Y. (2021). Antibacterial mechanism of apple phloretin on physiological and morphological properties of *Listeria monocytogenes*. Food Sci. Technol..

[42] Shuai-Cheng W., Ben-Dong F., Xiu-Ling C., Jian-Qing S., Yun-Xing F., Zhen-Qiang C., Dao-Xiu X., Zong-Mei W. (2016). Subinhibitory concentrations of phloretin repress the virulence of *Salmonella typhimurium* and protect against *Salmonella typhimurium* infection. Antonie Van Leeuwenhoek.

[43] Geohagen B.C., Korsharskyy B., Vydyanatha A., Nordstroem L., LoPachin R.M. (2018). Phloretin cytoprotection and toxicity. Chem. Biol. Interact..

[44] Londzin P., Siudak S., Cegieła U., Pytlik M., Janas A., Waligóra A., Folwarczna J. (2018). Phloridzin, an Apple Polyphenol, Exerted Unfavorable Effects on Bone and Muscle in an Experimental Model of Type 2 Diabetes in Rats. Nutrients.

[45] Gosch C., Halbwirth H., Stich K. (2010). Phloridzin: Biosynthesis, distribution and physiological relevance in plants. Phytochemistry.

[46] Zhao Y.Y., Fan Y., Wang M., Wang J., Cheng J.X., Zou J.B., Zhang X.F., Shi Y.J., Guo D.Y. (2020). Studies on pharmacokinetic properties and absorption mechanism of phloretin: In vivo and in vitro. Biomed. Pharmacother..

[47] Guo D., Liu J., Fan Y., Cheng J., Shi Y., Zou J., Zhang X. (2020). Optimization, characterization and evaluation of liposomes from *Malus hupehensis* (Pamp.) Rehd. extracts. J. Liposome Res..

[48] Sharifi-Rad A., Mehrzad J., Darroudi M., Saberi M.R., Chamani J. (2021). Oil-in-water nanoemulsions comprising Berberine in olive oil: Biological activities, binding mechanisms to human serum albumin or holo-transferrin and QMMD simulations. J. Biomol. Struct. Dyn..

[49] Remsberg C.M., Yáñez J.A., Vega-Villa K., Miranda N.D., Andrews P.K. (2010). HPLC-UV Analysis of Phloretin in Biological Fluids and Application to Pre-Clinical Pharmacokinetic Studies. J. Chromatogr. Sep. Technol..

[50] Zhang T., Lian J., Wang P., Xu Y., Wang Y., Wei X., Fan M. (2016). Purification and characterization of a novel phloretin-2′-O-glycosyltransferase favoring phloridzin biosynthesis. Sci. Rep..

[51] Wang Y., Li D., Lin H., Jiang S., Han L., Hou S., Lin S., Cheng Z., Bian W., Zhang X. (2020). Enhanced oral bioavailability and bioefficacy of phloretin using mixed polymeric modified self-nanoemulsions. Food Sci. Nutr..

[52] Karabulut S., Toprak M. (2020). Biophysical study of phloretin with human serum albumin in liposomes using spectroscopic methods. Eur. Biophys. J..

[53] Abu-Azzam O., Nasr M. (2020). In vitro anti-inflammatory potential of phloretin microemulsion as a new formulation for prospective treatment of vaginitis. Pharm. Dev. Technol..

[54] Shin S., Kum H., Ryu D., Kim M., Jung E., Park D. (2014). Protective Effects of a New Phloretin Derivative against UVB-Induced Damage in Skin Cell Model and Human Volunteers. Int. J. Mol. Sci..

[55] Pandey R.P., Li T.F., Kim E.-H., Yamaguchi T., Park Y.I., Kim J.S., Sohng J.K. (2013). Enzymatic Synthesis of Novel Phloretin Glucosides. Appl. Environ. Microbiol..

[56] Minsat L., Peyrot C., Brunissen F., Renault J.-H., Allais F. (2021). Synthesis of Biobased Phloretin Analogues: An Access to Antioxidant and Anti-Tyrosinase Compounds for Cosmetic Applications. Antioxidants.

[57] Wang L., Li Z.W., Zhang W., Xu R., Gao F., Liu Y.F., Li Y.J. (2014). Synthesis, Crystal Structure, and Biological Evaluation of a Series of Phloretin Derivatives. Molecules.

[58] Shimoda K., Otsuka T., Morimoto Y., Hamada H., Hamada H. (2007). Glycosylation and malonylation of quercetin, epicatechin, and catechin by cultured plant cells. Chem. Lett..

[59] Peerce B.E., Clarke R. (2022). A phosphorylated phloretin derivative. Synthesis and effect on intestinal Na ϩ-dependent phosphate absorption. Am. J. Physiol. Liver Physiol..

[60] Difdrich D.F. (1990). Photoafflnity-Labeling Analogs of Phlorizin and Phloretin: Synthesis and Effects on Cell Membranes. Methods Enzymol..

[61] Rana S., Bhushan S. (2016). Apple phenolics as nutraceuticals: Assessment, analysis and application. J. Food Sci. Technol..

[62] Shim S., Jo S., Kim J., Choi G.J. (2010). Control Efficacy of Phloretin Isolated from Apple Fruits Against Several Plant Diseases. Plant Pathol. J..

[63] Kindt M., Orsini M.C., Costantini B. (2007). Improved High-Performance Liquid Chromatography—Diode Array Detection Method for the Determination of Phenolic Compounds in Leaves and Peels from Different Apple Varieties. J. Chromatogr. Sci..

[64] Qin X., Xing Y., Zhou Z., Yao Y. (2015). Dihydrochalcone Compounds Isolated from Crabapple Leaves Showed Anticancer Effects on Human Cancer Cell Lines. Molecules.

[65] Ben-Othman S., Kaldmäe H., Rätsep R., Bleive U., Aluvee A., Rinken T. (2021). Optimization of ultrasound-assisted extraction of phloretin and other phenolic compounds from apple tree leaves (Malus domestica Borkh.) and comparison of different cultivars from Estonia. Antioxidants.

[66] Adamcová A., Horna A., Šatínský D. (2022). Determination of Phloridzin and Other Phenolic Compounds in Apple Tree Leaves, Bark, and Buds Using Liquid Chromatography with Multilayered Column Technology and Evaluation of the Total Antioxidant Activity. Pharmaceuticals.

[67] Petkovska A., Gjamovski V., Stanoeva J.P., Stefova M. (2017). Characterization of the Polyphenolic Profiles of Peel, Flesh and Leaves of Malus domestica Cultivars Using UHPLC-DAD-HESI-MS. Nat. Prod. Commun..

[68] Rana S., Rana A., Gulati A., Bhushan S. (2014). RP-HPLC-DAD Determination of Phenolics in Industrial Apple Pomace. Food Anal. Methods.

[69] Zhang T., Wei X., Miao Z., Hassan H., Song Y., Fan M. (2016). Screening for antioxidant and antibacterial activities of phenolics from Golden Delicious apple pomace. Chem. Cent. J..

[70] Lommen A., Godejohann M., Venema D.P., Hollman P.C.H., Spraul M. (2000). Application of Directly Coupled HPLC-NMR-MS to the Identification and Confirmation of Quercetin Glycosides and Phloretin Glycosides in Apple Peel. Anal. Chem..

[71] Preti R., Maria A. (2021). Study of polyphenols, antioxidant capacity and minerals for the valorisation of ancient apple cultivars from Northeast Italy. Eur. Food Res. Technol..

[72] Xü K., Lü H., Qü B., Shan H., Song J. (2010). High-speed counter-current chromatography preparative separation and purification of phloretin from apple tree bark. Sep. Purif. Technol..

[73] Hilt P., Schieber A., Yildirim C., Arnold G., Klaiber I., Conrad J., Beifuss U., Carle R. (2003). Detection of Phloridzin in Strawberries (Fragaria x ananassa Duch.) by HPLC − PDA − MS/MS and NMR Spectroscopy. J. Agric. Food Chem..

[74] Mehta D., Yadav S.K. (2020). Impact of atmospheric non-thermal plasma and hydrothermal treatment on bioactive compounds and microbial inactivation of strawberry juice: A hurdle technology approach. Food Sci. Technol. Int..

[75] Gasperotti M., Masuero D., Mattivi F., Vrhovsek U. (2014). Overall dietary polyphenol intake in a bowl of strawberries: The influence of *Fragaria* spp. in nutritional studies. J. Funct. Foods.

[76] Jaroslawska J., Juskiewicz J., Wroblewska M., Jurgonski A., Krol B. (2011). Polyphenol-Rich Strawberry Pomace Reduces Serum and Liver Lipids and Alters Gastrointestinal Metabolite Formation in Fructose-Fed Rats. J. Nutr. Nutr. Physiol. Metab. Nutr. Interact..

[77] Pubchem Pubchem (2022). PubChem Compd. Summ. CID 4788, Phloretin.

[78] Li B., Li R., Yan W. (2011). Solubilities of Phloretin in 12 Solvents at Different Temperatures. J. Chem. Eng. Data.

[79] Oresajo C., Stephens T., Hino P.D., Law R.M., Yatskayer M., Foltis P., Pillai S., Pinnell S.R. (2008). Protective effects of a topical antioxidant mixture containing vitamin C, ferulic acid, and phloretin against ultraviolet-induced photodamage in human skin. J. Cosmet. Dermatol..

[80] Crespy V., Aprikian O., Morand C., Besson C., Manach C., Demigné C., Rémésy C. (2001). Bioavailability of phloretin and phloridzin in rats. J. Nutr..

[81] Bentes A.L.A., Borges R.S., Monteiro W.R., De Macedo L.G.M., Alves C.N. (2011). Structure of dihydrochalcones and related derivatives and their scavenging and antioxidant activity against oxygen and nitrogen radical species. Molecules.

[82] Crespy V., Aprikian O., Morand C., Besson C., Manach C., Demigne C. (2002). Bioavailability of Phloretin and Phloridzin in Rats. Nutr. Metab. Commun..

[83] Behzad S., Sureda A., Barreca D., Fazel S., Rastrelli L., Mohammad S. (2017). Health effects of phloretin: From chemistry to medicine. Phytochem. Rev..

[84] Aitipamula S., Chow P.S., Tan R.B.H. (2014). Polymorphism in cocrystals: A review and assessment of its significance. CrystEngComm.

[85] Aitipamula S., Shan L.P., Gupta K.M. (2022). Polymorphism and distinct physicochemical properties of the phloretin–nicotinamide cocrystal. CrystEngComm.

[86] Hassanpour S.H., Dehghani M. (2017). Review of cancer from perspective of molecular. J. Cancer Res. Pract..

[87] Ranjan A., Ramachandran S., Gupta N., Kaushik I., Wright S., Srivastava S., Das H., Srivastava S., Prasad S., Srivastava S.K. (2019). Role of Phytochemicals in Cancer Prevention. Int. J. Mol. Sci..

[88] Mariadoss A.V.A., Vinyagam R., Rajamanickam V., Sankaran V., Venkatesan S., David E. (2019). Pharmacological Aspects and Potential Use of Phloretin: A Systemic Review. Mini Rev. Med. Chem..

[89] Kobori M., Shinmoto H., Tsushida T., Shinohara K. (1997). Phloretin-induced apoptosis in B16 melanoma 4A5 cells by inhibition of glucose transmembrane transport. Cancer Lett..

[90] Zhou M., Zheng J., Bi J., Wu X., Lyu J., Gao K. (2018). Synergistic inhibition of colon cancer cell growth by a combination of atorvastatin and phloretin. Oncol. Lett..

[91] Adinortey M.B., Agbeko R., Boison D., Ekloh W., Kuatsienu L.E., Biney E.E., Affum O.O., Kwarteng J., Nyarko A.K. (2019). Phytomedicines Used for Diabetes Mellitus in Ghana: A Systematic Search and Review of Preclinical and Clinical Evidence. Evid. Based. Complement. Alternat. Med..

[92] Arora S.K., Verma P.R., Itankar P.R., Prasad S.K., Nakhate K.T. (2021). Evaluation of pancreatic regeneration activity of Tephrosia purpurea leaves in rats with streptozotocin-induced diabetes. J. Tradit. Complement. Med..

[93] Ehrenkranz J.R.L., Lewis N.G., Ronald Kahn C., Roth J. (2005). Phlorizin: A review. Diabetes. Metab. Res. Rev..

[94] Sampath C., Rashid M.R., Sang S., Ahmedna M. (2017). Specific bioactive compounds in ginger and apple alleviate hyperglycemia in mice with high fat diet-induced obesity via Nrf2 mediated pathway. Food Chem..

[95] Shen X., Zhou N., Mi L., Hu Z., Wang L., Liu X., Zhang S. (2017). Phloretin exerts hypoglycemic effect in streptozotocin-induced diabetic rats and improves insulin resistance in vitro. Drug Des. Devel. Ther..

[96] Alsanea S., Gao M., Liu D. (2017). Phloretin Prevents High-Fat Diet-Induced Obesity and Improves Metabolic Homeostasis. AAPS J..

[97] Kumar V., Singh D.D., Lakhawat S.S., Yasmeen N., Pandey A., Singla R.K. (2022). Biogenic Phytochemicals Modulating Obesity: From Molecular Mechanism to Preventive and Therapeutic Approaches. Evidence-Based Complement. Altern. Med..

[98] Nakhate K.T., Dandekar M.P., Kokare D.M., Subhedar N.K. (2009). Involvement of neuropeptide Y Y1 receptors in the acute, chronic and withdrawal effects of nicotine on feeding and body weight in rats. Eur. J. Pharmacol..

[99] Casado-Díaz A., Rodríguez-Ramos Á., Torrecillas-Baena B., Dorado G., Quesada-Gómez J.M., Gálvez-Moreno M.Á. (2021). Flavonoid Phloretin Inhibits Adipogenesis and Increases OPG Expression in Adipocytes Derived from Human Bone-Marrow Mesenchymal Stromal-Cells. Nutrients.

[100] Thiriet M. (2019). Cardiovascular Disease: An Introduction. Vasc. Behav. Chem. Environ. Genet. Factors.

[101] Eberhardt M.V., Lee C.Y., Liu R.H. (2000). Antioxidant activity of fresh apples. Nature.

[102] Pawar H.D., Mahajan U.B., Nakhate K.T., Agrawal Y.O., Patil C.R., Meeran M.F., Sharma C., Ojha S., Goyal S.N. (2022). Curcumin Protects Diabetic Mice against Isoproterenol-Induced Myocardial Infarction by Modulating CB2 Cannabinoid Receptors. Life.

[103] Rathod S., Agrawal Y., Sherikar A., Nakhate K.T., Patil C.R., Nagoor Meeran M.F., Ojha S., Goyal S.N. (2022). Thymoquinone Produces Cardioprotective Effect in β-Receptor Stimulated Myocardial Infarcted Rats via Subsiding Oxidative Stress and Inflammation. Nutrients.

[104] Wagh S.S., Patil K.R., Mahajan U.B., Bagal P.D., Wadkar A.R., Bommanhalli B., Patil P.R., Goyal S.N., Ojha S., Patil C.R. (2022). Phloretin-induced suppression of oxidative and nitrosative stress attenuates doxorubicin-induced cardiotoxicity in rats. Asian Pac. J. Trop. Biomed..

[105] Sauri I., Uso R., Trillo J.L., Fernandez A., Holgado J.L., Lopez C., Vela S., Bea C., Ruiz A., Martinez F. (2021). Impact of hypertension in the morbidity and mortality in diabetes mellitus: A real-world data. J. Hypertens..

[106] Kaplan N.M. (2002). Hypertension and diabetes. J. Hum. Hypertens..

[107] Zhang Y.J., Gan R.Y., Li S., Zhou Y., Li A.N., Xu D.P., Li H.B. (2015). Antioxidant Phytochemicals for the Prevention and Treatment of Chronic Diseases. Molecules.

[108] Khichariya A., Jeswani G., Choudhary R., Alexander A., Nakhate K.T., Badwaik H.R. (2022). Formulation of Plumbagin Loaded Microemulsion: Evaluation of Anti-rheumatoid efficacy in Wistar Rat model. J. Mol. Liq..

[109] Wei Y., Zhang J., Memon A.H., Liang H. (2017). Molecular model and in vitro antioxidant activity of a water-soluble and stable phloretin/hydroxypropyl-β-cyclodextrin inclusion complex. J. Mol. Liq..

[110] Huang W.C., Dai Y.W., Peng H.L., Kang C.W., Kuo C.Y., Liou C.J. (2015). Phloretin ameliorates chemokines and ICAM-1 expression via blocking of the NF-κB pathway in the TNF-α-induced HaCaT human keratinocytes. Int. Immunopharmacol..

[111] Cui D., Liu S., Tang M., Lu Y., Zhao M., Mao R., Wang C., Yuan Y., Li L., Chen Y. (2020). Phloretin ameliorates hyperuricemia-induced chronic renal dysfunction through inhibiting NLRP3 inflammasome and uric acid reabsorption. Phytomedicine.

[112] Chang W.T., Huang W.C., Liou C.J. (2012). Evaluation of the anti-inflammatory effects of phloretin and phlorizin in lipopolysaccharide-stimulated mouse macrophages. Food Chem..

[113] Huang W.C., Lai C.L., Liang Y.T., Hung H.C., Liu H.C., Liou C.J. (2016). Phloretin attenuates LPS-induced acute lung injury in mice via modulation of the NF-κB and MAPK pathways. Int. Immunopharmacol..

[114] Nakhate K., Borkar C., Bharne A., Sinha G.R., Suri J.S. (2020). Chapter 2—Functional neuroanatomy and disorders of cognition. Cognitive Informatics, Computer Modelling, and Cognitive Science.

[115] Nakhate K.T., Bharne A.P., Verma V.S., Aru D.N., Kokare D.M. (2018). Plumbagin ameliorates memory dysfunction in streptozotocin induced Alzheimer’s disease via activation of Nrf2/ARE pathway and inhibition of β-secretase. Biomed. Pharmacother..

[116] Rangani R.J., Upadhya M.A., Nakhate K.T., Kokare D.M., Subhedar N.K. (2012). Nicotine evoked improvement in learning and memory is mediated through NPY Y1 receptors in rat model of Alzheimer’s disease. Peptides.

[117] Upadhya M.A., Nakhate K.T., Kokare D.M., Singru P.S., Subhedar N.K. (2011). Cocaine- and amphetamine-regulated transcript peptide increases spatial learning and memory in rats. Life Sci..

[118] Gothwal A., Kumar H., Nakhate K.T., Ajazuddin, Dutta A., Borah A., Gupta U. (2019). Lactoferrin Coupled Lower Generation PAMAM Dendrimers for Brain Targeted Delivery of Memantine in Aluminum-Chloride-Induced Alzheimer’s Disease in Mice. Bioconjug. Chem..

[119] Gothwal A., Nakhate K.T., Alexander A., Ajazuddin A., Gupta U. (2018). Boosted Memory and Improved Brain Bioavailability of Rivastigmine: Targeting Effort to the Brain Using Covalently Tethered Lower Generation PAMAM Dendrimers with Lactoferrin. Mol. Pharm..

[120] Ghumatkar P.J., Patil S.P., Jain P.D., Tambe R.M., Sathaye S. (2015). Nootropic, neuroprotective and neurotrophic effects of phloretin in scopolamine induced amnesia in mice. Pharmacol. Biochem. Behav..

[121] Ghumatkar P.J., Patil S.P., Peshattiwar V., Vijaykumar T., Dighe V., Vanage G., Sathaye S. (2019). The modulatory role of phloretin in Aβ25–35 induced sporadic Alzheimer’s disease in rat model. Naunyn. Schmiedebergs. Arch. Pharmacol..

[122] Ghumatkar P., Peshattiwar V., Patil S., Muke S., Whitfield D., Howlett D., Francis P., Sathaye S. (2018). The effect of phloretin on synaptic proteins and adult hippocampal neurogenesis in Aβ (1-42)-injected male Wistar rats. J. Pharm. Pharmacol..

[123] Zhang G., Yang G., Liu J. (2019). Phloretin attenuates behavior deficits and neuroinflammatory response in MPTP induced Parkinson’s disease in mice. Life Sci..

[124] Liu N., Zhang N., Zhang S., Zhang L., Liu Q. (2021). Phloretin inhibited the pathogenicity and virulence factors against Candida albicans. Bioengineered.

[125] Duan H., Wang R., Yan X., Liu H., Zhang Y., Mu D., Han J., Li X. (2017). Phloretin induces apoptosis of human esophageal cancer via a mitochondria dependent pathway. Oncol. Lett..

[126] Abkin S.V., Ostroumova O.S., Komarova E.Y., Meshalkina D.A., Shevtsov M.A., Margulis B.A., Guzhova I. (2016). V Phloretin increases the anti-tumor efficacy of intratumorally delivered heat-shock protein 70 kDa (HSP70) in a murine model of melanoma. Cancer Immunol. Immunother..

[127] Yang K., Tsai C., Wang Y., Wei P., Lee C., Chen J., Wu C., Ho Y. (2009). Apple polyphenol phloretin potentiates the anticancer actions of paclitaxel through induction of apoptosis in human hep G2 cells. Mol. Carcinog. Publ. Coop. with Univ. Texas MD Anderson Cancer Cent..

[128] Goyal S.N., Prajapati C.P., Gore P.R., Patil C.R., Mahajan U.B., Sharma C., Talla S.P., Ojha S.K. (2017). Therapeutic potential and pharmaceutical development of thymoquinone: A multitargeted molecule of natural origin. Front. Pharmacol..

[129] Badwaik H.R., Kumari L., Nakhate K., Verma V.S., Sakure K. (2019). Phytoconstituent plumbagin: Chemical, biotechnological and pharmaceutical aspects. Stud. Nat. Prod. Chem..

[130] Badwaik H., Giri T., Nakhate K., Kashyap P., Tripathi D. (2013). Xanthan Gum and Its Derivatives as a Potential Bio-polymeric Carrier for Drug Delivery System. Curr. Drug Deliv..

[131] Badwaik H.R., Nakhate K., Kumari L., Sakure K. (2018). Oral Delivery of Proteins and Polypeptides through Polysaccharide Nanocarriers. Polysaccharide-Based Nano-Biocarrier in Drug Delivery.

[132] Khan I., Joshi G., Sarkar B., Nakhate K.T., Ajazuddin, Mantha A.K., Kumar R., Kaul A., Chaturvedi S., Mishra A.K. (2020). Doxorubicin and Crocin Co-delivery by Polymeric Nanoparticles for Enhanced Anticancer Potential in Vitro and in Vivo. ACS Appl. Bio Mater..

[133] Badwaik H.R., Kumari L., Maiti S., Sakure K., Nakhate K.T., Tiwari V., Giri T.K. (2022). A review on challenges and issues with carboxymethylation of natural gums: The widely used excipients for conventional and novel dosage forms. Int. J. Biol. Macromol..

[134] Chen Y., Xue J., Luo Y. (2020). Encapsulation of Phloretin in a Ternary Nanocomplex Prepared with Phytoglycogen–Caseinate–Pectin via Electrostatic Interactions and Chemical Cross-Linking. J. Agric. Food Chem..

[135] Mariadoss A.V.A., Vinayagam R., Senthilkumar V., Paulpandi M., Murugan K., Xu B., Gothandam K.M., Kotakadi V.S., David E. (2019). Phloretin loaded chitosan nanoparticles augments the pH-dependent mitochondrial-mediated intrinsic apoptosis in human oral cancer cells. Int. J. Biol. Macromol..

[136] Gu L., Sun R., Wang W., Xia Q. (2022). Nanostructured lipid carriers for the encapsulation of phloretin: Preparation and in vitro characterization studies. Chem. Phys. Lipids.

[137] Casarini T.P.A., Frank L.A., Benin T., Onzi G., Pohlmann A.R., Guterres S.S. (2021). Innovative hydrogel containing polymeric nanocapsules loaded with phloretin: Enhanced skin penetration and adhesion. Mater. Sci. Eng. C.

[138] Nam S., Lee S.Y., Cho H.-J. (2017). Phloretin-loaded fast dissolving nanofibers for the locoregional therapy of oral squamous cell carcinoma. J. Colloid Interface Sci..

[139] Ranjanamala T., Vanmathiselvi K., Casimeer S.C., Ghidan A.Y. (2022). Synthesis and Characterization of Dose-Dependent Antioxidants and Antimicrobial Activity of Phloretin Loaded PLGA Nanoparticles. Biointerface Res. Appl. Chem..

[140] Payne J.N., Badwaik V.D., Waghwani H.K., Moolani H.V., Tockstein S., Thompson D.H., Rajalingam D. (2018). Development of dihydrochalcone-functionalized gold nanoparticles for augmented antineoplastic activity. Int. J. Nanomed..

[141] Yang J.Z., Zhao X.Y., Zhou K., Zhang Z.Q., Xing-Ye Z. (2021). Formulation optimization of phloretin nanostructured lipid carrier by Box-Behnken design-response surface method. Chinese J. Hosp. Pharm..

[142] Kum H., Roh K.-B., Shin S., Jung K., Park D., Jung E. (2016). Evaluation of anti-acne properties of phloretin in vitro and in vivo. Int. J. Cosmet. Sci..

[143] Pinnell S.R., Zielinski J., Hansenne I. (2016). Anti-Aging Composition Containing Phloretin. Patent.

[144] Hu X., Zhou Z., Han L., Li S., Zhou W. (2020). Preparation and characterization of phloretin by complexation with cyclodextrins. New J. Chem..

